# Crystal structure of *meso*-3,3′-(1,4-phenyl­ene)bis­(2-phenyl-2,3,5,6-tetra­hydro-4*H*-1,3-thia­zin-4-one)

**DOI:** 10.1107/S2056989018013397

**Published:** 2018-09-28

**Authors:** Hemant P. Yennawar, Quentin J. Moyer, Lee J. Silverberg

**Affiliations:** a Department of Biochemistry and Molecular Biology, Pennsylvania State University, University Park PA 16802 , USA; b Pennsylvania State University, Schuylkill Campus, 200 University Drive, Schuylkill Haven, PA 17972, USA

**Keywords:** crystal structure, bis-thia­zinone, C—H⋯O and C—H⋯π inter­actions, *meso* structure

## Abstract

The crystal structure of the title compound – *meso*-C_26_H_24_N_2_O_2_S_2_ with two stereocenters – has half the mol­ecule in the asymmetric unit with the other half generated by a crystallographic center of inversion. The thia­zine ring is in a conformation that is between half-chair and envelope [θ = 52.51 (17)°]. The phenyl ring on the 2-carbon atom of the thia­zine ring is pseudo-axial. The central phenyl ring of the mol­ecule is close to orthogonal to the phenyl rings on either side with an angle of 76.85 (11)° between those planes. In the crystal, pairwise, weak C—H⋯O hydrogen bonds between the central phenyl ring and the oxygen atoms of neighboring mol­ecules result in continuous strips propagating along the *a*-axis direction. Hydro­phobic inter­actions of the C—H⋯π type are also observed.

## Abstract 2

The complete mol­ecule of the title phenyl­ene-bridged bis-heterocycle, a *meso* compound, is generated by a crystallographic centre of symmetry.

## Chemical context   

Bis-heterocyclic compounds are of inter­est because of their potential biological activity (Shaker, 2012 ▸). The phenyl­ene bridged bis-thia­zolidinone 3,3′-(1,4-phenyl­ene)bis­(2-phenyl-l,3-thia­zolidin-4-one) has been reported by multiple groups over several decades (Martani, 1956 ▸; El-Shafei *et al.*, 1984 ▸; Shaker, 1999 ▸; Kumar *et al.*, 2013 ▸; Pang *et al.*, 2016 ▸; Xing *et al.*, 2016 ▸), but the analogous bis-2,3,5,6-tetra­hydro-4*H*-1,3-thia­zin-4-one has not. There is a report of 3,3′-(1,4-phenyl­ene)bis­(2-(4-methyl­phen­yl)-2,3,5,6-tetra­hydro-4*H*-1,3-thia­zin-4-one), but the data supporting the assigned structure are questionable (Aljamali, 2013 ▸). There do not appear to be any other reports of a 3,3′-(1,4-phenyl­ene)bis­(2-ar­yl-2,3,5,6-tetra­hydro-4*H*-1,3-thia­zin-4-one). In previous work, we have reported the synthesis and crystal structures of several mono-heterocyclic 2,3-diaryl-2,3,5,6-tetra­hydro-4*H*-1,3-thia­zin-4-ones (Yennawar & Silverberg, 2014 ▸, 2015 ▸; Yennawar *et al.*, 2018 ▸). Herein we report the synthesis and crystal structure of *meso*-3,3′-(1,4-phenyl­ene)bis­(2-phenyl-2,3,5,6-tetra­hydro-4*H*-1,3-thia­zin-4-one), (I). There are two stereocenters in the mol­ecule, at the 2-C position of each heterocycle, but the only stereoisomer isolated was the *meso* structure, *i.e.* the stereocenters have opposite configurations.
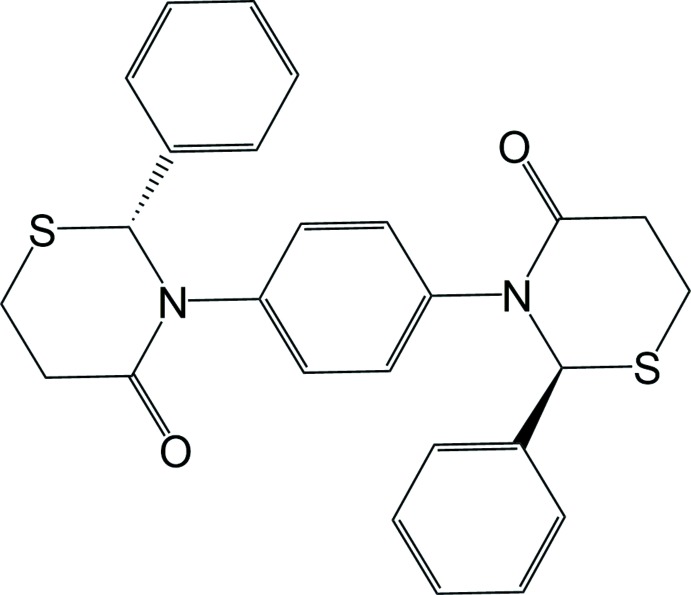


## Structural commentary   

**Figure 1:**
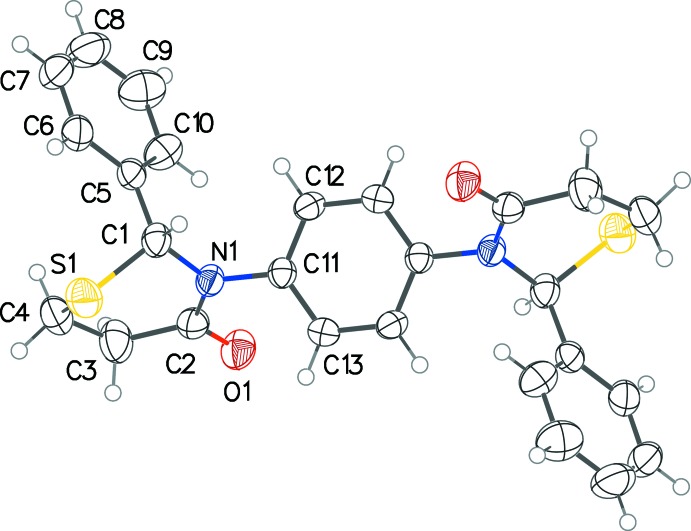
The mol­ecular structure of (I) with displacement ellipsoids drawn at the 50% probability level. The asymmetric unit contains half the mol­ecule (unique atoms shown with labels); the unlabeled atoms are generated by the symmetry operation (2 − *x*, 1 − *y*, −*z*).

**Figure 2:**
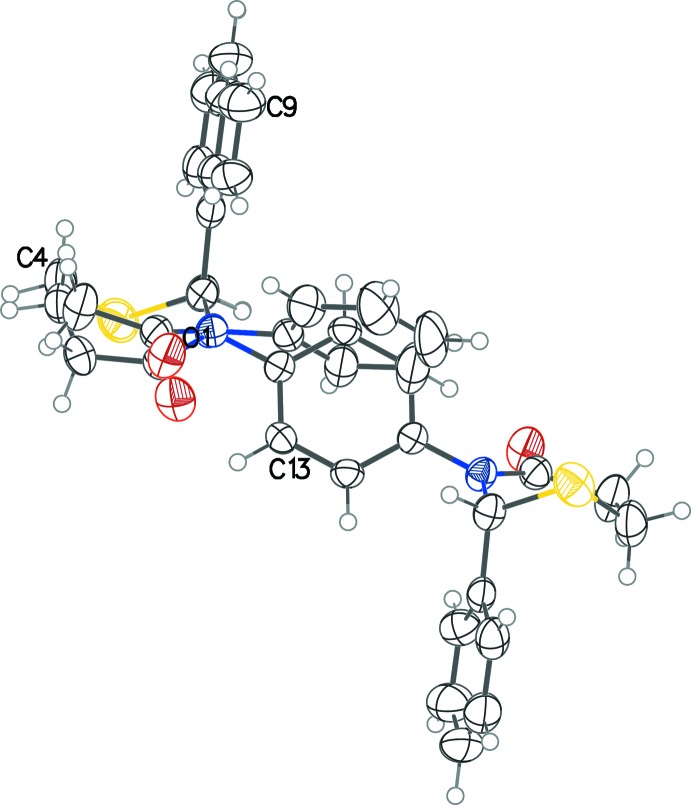
Overlay image of the title mol­ecule (a few atoms labeled) with 2,3-diphenyl-2,3,5,6-tetra­hydro-4*H*-1,3-thia­zin-4-one (Yennawar & Silverberg, 2014 ▸) showing differences in the central ring orientation in the two structures.

Compound (I) is highly symmetric with two chiral centers and its *meso* stereochemistry allows it to straddle the center of inversion in the *P*2_1_/*c* space-group (Fig. 1 ▸). The thia­zine rings adopt a configuration midway between half-chair and envelope [θ = 52.51 (17)°], with the sulfur atoms in each forming the back or the flap. On each thia­zine ring, the phenyl group on the 2-carbon atom is pseudo-axial. The dihedral angle between the planes of the two substituent phenyl rings is 76.85 (11)°. The structure described above shows some similarities and some differences when compared with that of 2,3-diphenyl-2,3,5,6-tetra­hydro-4*H*-1,3-thia­zin-4-one, (II) (Yennawar & Silverberg, 2014 ▸). In (II), the thia­zine ring has an envelope conformation [θ = 54.54 (17)°] and the orientation of the phenyl ring on the 3-nitro­gen atom about the N—C bond differs by about 90° from the structure of (I), as can be seen in superposition image (Fig. 2 ▸).

## Supra­molecular features   

**Figure 3:**
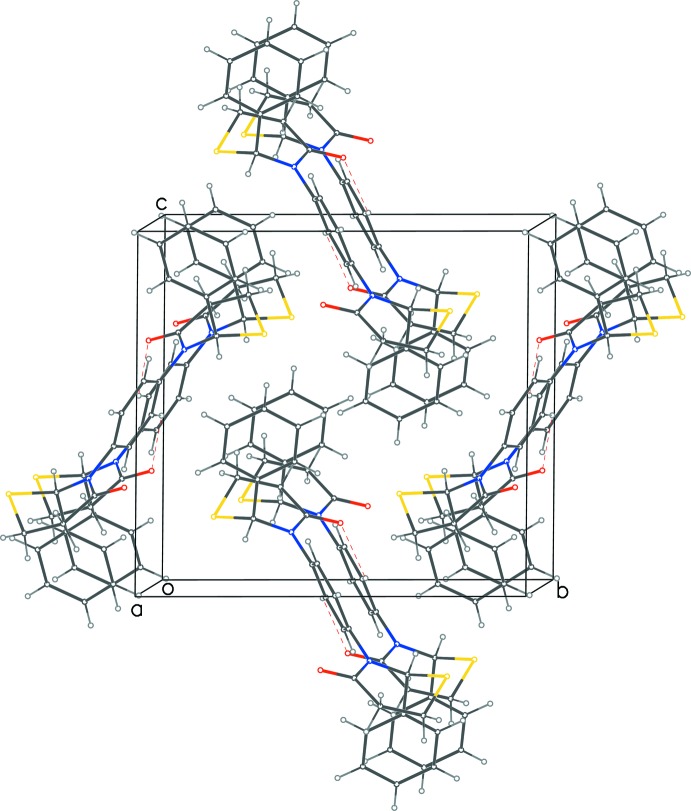
Packing diagram for (I) showing continuous tape formations linked by weak C—H⋯O inter­actions (dashed lines) propagating along the [100] direction.

A very weak C—H⋯O hydrogen bond between the central phenyl ring and the oxygen atom of the neighboring mol­ecule is detailed in Table 1 ▸. In the extended structure, these hydrogen bonds result in parallel and reciprocal pairs of inter­actions, which further give rise to a pair of continuous tape formations down the *a-*axis direction (Fig. 3 ▸), defined by the lines (*x*, ½, 0) and (*x*, 0, ½). In addition, a C—H⋯π inter­action [C⋯π-ring = 3.457 (3) Å] between the carbon atom of the thia­zine ring and the 2-phenyl ring is observed.

## Database survey   

The crystal structure of the mono-heterocycle 2,3-diphenyl-2,3,5,6-tetra­hydro-4*H*-1,3-thia­zin-4-one (Yennawar & Silverberg, 2014 ▸) was the closest crystal structure found. Similarity and substructure searches on SciFinder, repeated 9/25/18, only found one phenylene-bridged bis-(1,3-thiazin-4-one) compound, which almost certainly was incorrectly identified (Aljamali, 2013 ▸). No crystal structures of this or phenylene-bridged bis-(1,3-thiazolidin-4-one) compounds were found either.

## Synthesis and crystallization   

***meso*** **-3,3′-(1,4-Phenyl­ene)bis­(2-phenyl-2,3,5,6-tetra­hydro-4** ***H*** **-1,3-thia­zin-4-one):** A two-necked 25-ml round-bottom flask was oven-dried, cooled under N_2_, and charged with a stir bar, *N*,*N*′-(1,4-phenyl­ene)bis­(1-phenyl­methanimine) (0.8531 g, 3 mmol) and 3-mercaptopropionic acid (0.6368 g, 6 mmol). 2-Methyl­tetra­hydro­furan (2.3 ml) was added and the solution was stirred. Pyridine (1.95 ml, 24 mmol) and finally, 2,4,6-tri­propyl-1,3,5,2,4,6-trioxatri­phospho­rinane-2,4,6-trioxide (T3P) in 2-methyl­tetra­hydro­furan (50 weight %; 7.3 ml, 12 mmol) were added. The reaction was stirred at room temperature and followed by TLC (80% ethyl acetate/hexa­nes). The mixture was poured into a separatory funnel with di­chloro­methane and distilled water. The layers were separated and the aqueous layer was then extracted twice with di­chloro­methane. The organics were combined and washed with saturated sodium bicarbonate and then saturated sodium chloride. The organic was dried over sodium sulfate and concentrated under vacuum to give crude product. The crude was recrystallized from CH_2_Cl_2_/acetone solution to give white powder. Yield: 0.3108 g 1st crop, 0.0318 g 2nd crop (12% total), m.p. 523 K (decomp.). Crystals suitable for X-ray diffraction studies were grown by slow evaporation from CH_2_Cl_2_/acetone.

## Refinement   

Crystal data, data collection and structure refinement details are summarized in Table 2 ▸. H atoms were positioned geometerically (C—H = 0.93–0.98 Å) and refined as riding with *U*_iso_(H) = 1.2*U*_eq_(C).

## Supplementary Material

Crystal structure: contains datablock(s) I. DOI: 10.1107/S2056989018013397/hb7772sup1.cif

Structure factors: contains datablock(s) I. DOI: 10.1107/S2056989018013397/hb7772Isup2.hkl

Supporting information file. DOI: 10.1107/S2056989018013397/hb7772Isup3.mol

CCDC reference: 1868690

Additional supporting information:  crystallographic information; 3D view; checkCIF report

## Figures and Tables

**Table 1:** Hydrogen-bond geometry (Å, °)

*D*—H⋯*A*	*D*—H	H⋯*A*	*D*⋯*A*	*D*—H⋯*A*
C13—H13⋯O1^i^	0.93	2.72	3.401 (3)	131

Symmetry code: (i) 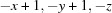.

**Table 2:** Experimental details

Crystal data	
Chemical formula	C_26_H_24_N_2_O_2_S_2_
*M*_r_	460.59
Crystal system, space group	Monoclinic, *P*2_1_/*c*
Temperature (K)	298
*a*, *b*, *c* (Å)	7.080 (2), 13.017 (4), 12.093 (3)
β (°)	98.289 (6)
*V* (Å^3^)	1102.9 (5)
*Z*	2
Radiation type	Mo *K*α
μ (mm^−1^)	0.27
Crystal size (mm)	0.15 × 0.06 × 0.05

Data collection	
Diffractometer	Bruker SMART CCD area detector
Absorption correction	Multi-scan (*SADABS*; Bruker, 2001 ▸4)
*T*_min_, *T*_max_	0.857, 0.9
No. of measured, independent and observed [*I* > 2σ(*I*)] reflections	7575, 2757, 2055
*R*_int_	0.023
(sin θ/λ)_max_ (Å^−1^)	0.668

Refinement	
*R*[*F* ^2^ > 2σ(*F* ^2^)], *wR*(*F* ^2^), *S*	0.056, 0.149, 1.02
No. of reflections	2757
No. of parameters	145
H-atom treatment	H-atom parameters constrained
Δρ_max_, Δρ_min_ (e Å^−3^)	0.29, −0.21

Computer programs: *SMART* and *SAINT* (Bruker, 2001 ▸), *SHELXS* and *SHELXL* (Sheldrick, 2008 ▸) and *OLEX2* (Dolomanov *et al.*, 2009 ▸).

## supplementary crystallographic information

### Crystal data

**Table:**

C_26_H_24_N_2_O_2_S_2_	*F*(000) = 484
*M**_r_* = 460.59	*D*_x_ = 1.387 Mg m^−^^3^
Monoclinic, *P*2_1_/*c*	Mo *K*α radiation, λ = 0.71073 Å
*a* = 7.080 (2) Å	Cell parameters from 1747 reflections
*b* = 13.017 (4) Å	θ = 2.3–26.2°
*c* = 12.093 (3) Å	µ = 0.27 mm^−^^1^
β = 98.289 (6)°	*T* = 298 K
*V* = 1102.9 (5) Å^3^	Block, colorless
*Z* = 2	0.15 × 0.06 × 0.05 mm

### Data collection

**Table:**

Bruker SMART CCD area detector diffractometer	2757 independent reflections
Radiation source: fine-focus sealed tube	2055 reflections with *I* > 2σ(*I*)
Graphite monochromator	*R*_int_ = 0.023
phi and ω scans	θ_max_ = 28.4°, θ_min_ = 2.3°
Absorption correction: multi-scan (SADABS; Bruker, 20014)	*h* = −9→9
*T*_min_ = 0.857, *T*_max_ = 0.9	*k* = −16→17
7575 measured reflections	*l* = −16→12

### Refinement

**Table:**

Refinement on *F*^2^	Primary atom site location: structure-invariant direct methods
Least-squares matrix: full	Secondary atom site location: difference Fourier map
*R*[*F*^2^ > 2σ(*F*^2^)] = 0.056	Hydrogen site location: inferred from neighbouring sites
*wR*(*F*^2^) = 0.149	H-atom parameters constrained
*S* = 1.02	*w* = 1/[σ^2^(*F*_o_^2^) + (0.0717*P*)^2^ + 0.4464*P*] where *P* = (*F*_o_^2^ + 2*F*_c_^2^)/3
2757 reflections	(Δ/σ)_max_ < 0.001
145 parameters	Δρ_max_ = 0.29 e Å^−^^3^
0 restraints	Δρ_min_ = −0.21 e Å^−^^3^

### Special details

**Table:**

Experimental. The data collection nominally covered a full sphere of reciprocal space by a combination of 4 sets of ω scans each set at different φ and/or 2θ angles and each scan (10 s exposure) covering -0.300° degrees in ω. The crystal to detector distance was 5.82 cm. SADABS V2.05 (BRUKER, 2001) was used for absorption correction. R(int) was 0.0303 before and 0.0175 after correction. The Ratio of minimum to maximum transmission is 0.8572. The λ/2 correction factor is 0.0015.
Geometry. All esds (except the esd in the dihedral angle between two l.s. planes) are estimated using the full covariance matrix. The cell esds are taken into account individually in the estimation of esds in distances, angles and torsion angles; correlations between esds in cell parameters are only used when they are defined by crystal symmetry. An approximate (isotropic) treatment of cell esds is used for estimating esds involving l.s. planes.
Refinement. Refinement of F^2^ against ALL reflections. The weighted R-factor wR and goodness of fit S are based on F^2^, conventional R-factors R are based on F, with F set to zero for negative F^2^. The threshold expression of F^2^ > 2sigma(F^2^) is used only for calculating R-factors(gt) etc. and is not relevant to the choice of reflections for refinement. R-factors based on F^2^ are statistically about twice as large as those based on F, and R- factors based on ALL data will be even larger.

### Fractional atomic coordinates and isotropic or equivalent isotropic displacement parameters (Å^2^)

**Table:**

	*x*	*y*	*z*	*U*_iso_*/*U*_eq_	
C1	0.9688 (3)	0.29893 (15)	0.21227 (18)	0.0388 (5)	
H1	1.0585	0.2852	0.1594	0.047*	
C2	0.6918 (3)	0.41602 (17)	0.20879 (17)	0.0392 (5)	
C3	0.5997 (4)	0.3422 (2)	0.2815 (2)	0.0588 (7)	
H3A	0.5923	0.3776	0.3514	0.071*	
H3B	0.4694	0.3322	0.2457	0.071*	
C4	0.6802 (4)	0.2374 (2)	0.3114 (2)	0.0601 (7)	
H4A	0.5760	0.1906	0.3187	0.072*	
H4B	0.7608	0.2410	0.3833	0.072*	
C5	1.0882 (3)	0.31690 (16)	0.32526 (18)	0.0387 (5)	
C6	1.1926 (3)	0.2367 (2)	0.3796 (2)	0.0523 (6)	
H6	1.1855	0.1714	0.3480	0.063*	
C7	1.3066 (3)	0.2534 (3)	0.4800 (2)	0.0654 (8)	
H7	1.3765	0.1993	0.5156	0.078*	
C8	1.3185 (4)	0.3491 (3)	0.5283 (2)	0.0739 (9)	
H8	1.3950	0.3597	0.5965	0.089*	
C9	1.2166 (4)	0.4288 (3)	0.4751 (2)	0.0675 (8)	
H9	1.2248	0.4939	0.5069	0.081*	
C10	1.1016 (3)	0.4123 (2)	0.37427 (19)	0.0502 (6)	
H10	1.0321	0.4668	0.3390	0.060*	
C11	0.9278 (3)	0.44770 (14)	0.08461 (15)	0.0317 (4)	
C12	1.1198 (3)	0.47343 (16)	0.09557 (16)	0.0350 (4)	
H12	1.2007	0.4556	0.1602	0.042*	
C13	0.8087 (3)	0.47470 (15)	−0.01124 (16)	0.0347 (4)	
H13	0.6798	0.4579	−0.0191	0.042*	
N1	0.8566 (2)	0.38859 (13)	0.16985 (14)	0.0355 (4)	
O1	0.6121 (2)	0.49719 (13)	0.18357 (15)	0.0539 (4)	
S1	0.81637 (9)	0.18725 (5)	0.21024 (6)	0.0574 (2)	

### Atomic displacement parameters (Å^2^)

**Table:**

	*U*^11^	*U*^22^	*U*^33^	*U*^12^	*U*^13^	*U*^23^
C1	0.0399 (10)	0.0339 (11)	0.0440 (11)	0.0052 (8)	0.0106 (8)	0.0067 (9)
C2	0.0346 (9)	0.0437 (12)	0.0396 (11)	0.0021 (9)	0.0059 (8)	0.0043 (9)
C3	0.0560 (14)	0.0589 (16)	0.0668 (16)	0.0056 (12)	0.0269 (12)	0.0170 (13)
C4	0.0485 (13)	0.0575 (16)	0.0759 (17)	−0.0081 (11)	0.0139 (12)	0.0209 (13)
C5	0.0313 (9)	0.0442 (12)	0.0422 (11)	0.0011 (8)	0.0103 (8)	0.0112 (9)
C6	0.0422 (11)	0.0492 (14)	0.0660 (15)	0.0022 (10)	0.0095 (11)	0.0201 (12)
C7	0.0426 (12)	0.085 (2)	0.0676 (17)	0.0072 (13)	0.0034 (12)	0.0370 (16)
C8	0.0538 (15)	0.116 (3)	0.0478 (15)	−0.0035 (16)	−0.0057 (12)	0.0142 (16)
C9	0.0668 (17)	0.081 (2)	0.0535 (15)	0.0025 (15)	0.0042 (13)	−0.0116 (14)
C10	0.0485 (12)	0.0545 (15)	0.0468 (12)	0.0104 (11)	0.0039 (10)	0.0014 (11)
C11	0.0334 (9)	0.0291 (10)	0.0329 (9)	0.0018 (7)	0.0064 (7)	0.0013 (7)
C12	0.0323 (9)	0.0405 (11)	0.0310 (9)	0.0029 (8)	−0.0002 (7)	0.0019 (8)
C13	0.0294 (8)	0.0371 (11)	0.0371 (10)	−0.0014 (7)	0.0031 (7)	−0.0012 (8)
N1	0.0342 (8)	0.0359 (9)	0.0373 (8)	0.0054 (7)	0.0085 (6)	0.0083 (7)
O1	0.0471 (8)	0.0525 (10)	0.0655 (10)	0.0173 (7)	0.0191 (8)	0.0169 (8)
S1	0.0605 (4)	0.0356 (3)	0.0744 (5)	−0.0044 (3)	0.0041 (3)	0.0002 (3)

### Geometric parameters (Å, º)

**Table:**

C1—H1	0.9800	C6—C7	1.375 (4)
C1—C5	1.518 (3)	C7—H7	0.9300
C1—N1	1.462 (2)	C7—C8	1.373 (5)
C1—S1	1.809 (2)	C8—H8	0.9300
C2—C3	1.512 (3)	C8—C9	1.370 (4)
C2—N1	1.367 (3)	C9—H9	0.9300
C2—O1	1.216 (3)	C9—C10	1.382 (3)
C3—H3A	0.9700	C10—H10	0.9300
C3—H3B	0.9700	C11—C12	1.388 (3)
C3—C4	1.502 (4)	C11—C13	1.377 (3)
C4—H4A	0.9700	C11—N1	1.435 (2)
C4—H4B	0.9700	C12—H12	0.9300
C4—S1	1.787 (3)	C12—C13^i^	1.379 (3)
C5—C6	1.389 (3)	C13—C12^i^	1.379 (3)
C5—C10	1.374 (3)	C13—H13	0.9300
C6—H6	0.9300		
			
C5—C1—H1	106.6	C7—C6—H6	119.9
C5—C1—S1	112.95 (14)	C6—C7—H7	119.6
N1—C1—H1	106.6	C8—C7—C6	120.8 (2)
N1—C1—C5	113.37 (17)	C8—C7—H7	119.6
N1—C1—S1	110.10 (13)	C7—C8—H8	120.3
S1—C1—H1	106.6	C9—C8—C7	119.4 (3)
N1—C2—C3	119.70 (19)	C9—C8—H8	120.3
O1—C2—C3	118.33 (19)	C8—C9—H9	120.0
O1—C2—N1	121.92 (19)	C8—C9—C10	120.1 (3)
C2—C3—H3A	106.7	C10—C9—H9	120.0
C2—C3—H3B	106.7	C5—C10—C9	121.0 (2)
H3A—C3—H3B	106.6	C5—C10—H10	119.5
C4—C3—C2	122.4 (2)	C9—C10—H10	119.5
C4—C3—H3A	106.7	C12—C11—N1	120.09 (16)
C4—C3—H3B	106.7	C13—C11—C12	119.42 (17)
C3—C4—H4A	109.0	C13—C11—N1	120.40 (17)
C3—C4—H4B	109.0	C11—C12—H12	119.8
C3—C4—S1	113.01 (18)	C13^i^—C12—C11	120.44 (17)
H4A—C4—H4B	107.8	C13^i^—C12—H12	119.8
S1—C4—H4A	109.0	C11—C13—C12^i^	120.14 (17)
S1—C4—H4B	109.0	C11—C13—H13	119.9
C6—C5—C1	120.0 (2)	C12^i^—C13—H13	119.9
C10—C5—C1	121.50 (18)	C2—N1—C1	122.35 (17)
C10—C5—C6	118.5 (2)	C2—N1—C11	120.86 (16)
C5—C6—H6	119.9	C11—N1—C1	116.80 (15)
C7—C6—C5	120.2 (3)	C4—S1—C1	94.42 (11)
			
C1—C5—C6—C7	177.6 (2)	C12—C11—N1—C2	134.4 (2)
C1—C5—C10—C9	−177.4 (2)	C13—C11—C12—C13^i^	−0.2 (3)
C2—C3—C4—S1	−26.3 (3)	C13—C11—N1—C1	131.14 (19)
C3—C2—N1—C1	−11.9 (3)	C13—C11—N1—C2	−49.0 (3)
C3—C2—N1—C11	168.2 (2)	N1—C1—C5—C6	175.76 (18)
C3—C4—S1—C1	53.0 (2)	N1—C1—C5—C10	−6.6 (3)
C5—C1—N1—C2	−77.3 (2)	N1—C1—S1—C4	−64.88 (16)
C5—C1—N1—C11	102.54 (19)	N1—C2—C3—C4	−1.4 (4)
C5—C1—S1—C4	62.97 (17)	N1—C11—C12—C13^i^	176.39 (18)
C5—C6—C7—C8	0.3 (4)	N1—C11—C13—C12^i^	−176.38 (18)
C6—C5—C10—C9	0.2 (3)	O1—C2—C3—C4	176.1 (3)
C6—C7—C8—C9	−0.5 (4)	O1—C2—N1—C1	170.7 (2)
C7—C8—C9—C10	0.6 (4)	O1—C2—N1—C11	−9.2 (3)
C8—C9—C10—C5	−0.5 (4)	S1—C1—C5—C6	49.6 (2)
C10—C5—C6—C7	−0.1 (3)	S1—C1—C5—C10	−132.72 (19)
C12—C11—C13—C12^i^	0.2 (3)	S1—C1—N1—C2	50.3 (2)
C12—C11—N1—C1	−45.4 (2)	S1—C1—N1—C11	−129.85 (15)

Symmetry code: (i) −*x*+2, −*y*+1, −*z*.

### Hydrogen-bond geometry (Å, º)

**Table:**

*D*—H···*A*	*D*—H	H···*A*	*D*···*A*	*D*—H···*A*
C13—H13···O1^ii^	0.93	2.72	3.401 (3)	131

Symmetry code: (ii) −*x*+1, −*y*+1, −*z*.
